# A monolithically integrated microcantilever biosensor based on partially depleted SOI CMOS technology

**DOI:** 10.1038/s41378-023-00534-y

**Published:** 2023-05-16

**Authors:** Yi Liu, Yuan Tian, Cong Lin, Jiahao Miao, Xiaomei Yu

**Affiliations:** School of Integrated Circuits, Peking University, National Key Laboratory of Science and Technology on Micro/Nano Fabrication, Beijing, 100871 China

**Keywords:** Electrical and electronic engineering, Biosensors

## Abstract

This paper presents a monolithically integrated aptasensor composed of a piezoresistive microcantilever array and an on-chip signal processing circuit. Twelve microcantilevers, each of them embedded with a piezoresistor, form three sensors in a Wheatstone bridge configuration. The on-chip signal processing circuit consists of a multiplexer, a chopper instrumentation amplifier, a low-pass filter, a sigma-delta analog-to-digital converter, and a serial peripheral interface. Both the microcantilever array and the on-chip signal processing circuit were fabricated on the single-crystalline silicon device layer of a silicon-on-insulator (SOI) wafer with partially depleted (PD) CMOS technology followed by three micromachining processes. The integrated microcantilever sensor makes full use of the high gauge factor of single-crystalline silicon to achieve low parasitic, latch-up, and leakage current in the PD-SOI CMOS. A measured deflection sensitivity of 0.98 × 10^−^^6^ nm^−1^ and an output voltage fluctuation of less than 1 μV were obtained for the integrated microcantilever. A maximum gain of 134.97 and an input offset current of only 0.623 nA were acquired for the on-chip signal processing circuit. By functionalizing the measurement microcantilevers with a biotin-avidin system method, human IgG, abrin, and staphylococcus enterotoxin B (SEB) were detected at a limit of detection (LOD) of 48 pg/mL. Moreover, multichannel detection of the three integrated microcantilever aptasensors was also verified by detecting SEB. All these experimental results indicate that the design and process of monolithically integrated microcantilevers can meet the requirements of high-sensitivity detection of biomolecules.

## Introduction

Since Binning et al. invented the atomic force microscope to investigate the surface of insulators on an atomic scale in 1986, microcantilever-based detection tools have gradually emerged^1^. To date, label-free microcantilever sensors with high sensitivity and small size have been successfully applied to the detection of various targeted molecules, such as DNA^2,3^, proteins^4–6^, viruses^7,8^, heavy metal ions^9,10^, and bacteria^11–13^. The detections were typically performed by measuring a frequency shift or a minuscule deflection induced by molecular adsorption, known as the “dynamic mode” and “static mode,” respectively. In general, microcantilevers operating in dynamic mode can achieve an extremely low limit of detection (LOD)^14–17^. However, the sensitivity of the microcantilevers significantly decreases in viscous environments due to the damping effect, which greatly limits their application in such conditions, including liquid environments^18^. To monitor the motion of microcantilevers in static or dynamic modes, a signal readout system is crucial, and readout schemes commonly use optical or electrical systems. Although optical readout can achieve extremely low LOD, the requisite sophisticated alignment leads to limited commercial availability. Piezoresistive microcantilevers provide an alternative technique to convert a small resistance change into a differential voltage signal using embedded piezoresistors, avoiding cumbersome optical readout equipment and facilitating monolithic integration with integrated circuits. In 2017, Zhao et al. applied a silicon-based piezoresistive microcantilever to detect DMMP with an LOD of 50 nM in PBS buffer^19^. In 2019, Li et al. reported a microcantilever aptasensor for detecting the tumor marker Mucin 1 with an LOD of 0.9 nM^20^. In 2019, Li et al. proposed a piezoresistive microcantilever sensor for the detection of gentamicin with an LOD of 9.44 μg/mL^21^.

For an electrical readout microcantilever sensor, it is attractive to monolithically integrate microcantilevers with a signal processing circuit, which allows for on-chip signal collection, signal amplification, signal conversion, and signal communication to enhance the overall performance of the sensor. Several groups have carried out research on monolithically integrated microcantilevers^22–28^. In 2002, Hagleitner et al. developed a piezoresistive microcantilever sensor monolithically integrated with a fully differential difference amplifier and achieved the detection of ethanol and toluene with LODs of 10 ppm and 1 ppm^26^. In 2013, Huang et al. reported a fully integrated microcantilever sensor and achieved the detection of DNA from hepatitis B virus at concentrations as low as 1 pM^27^. In 2014, Wei et al. presented a monolithic airflow detection chip composed of three microcantilever sensors and CMOS signal processing circuits^28^. However, the above integrated microcantilevers either operate in dynamic mode or were fabricated by bulk silicon CMOS processes, using polysilicon as a piezoresistor with a low piezoresistive coefficient.

In this paper, we propose monolithically integrated microcantilever aptasensors based on partially depleted (PD) silicon-on-insulator (SOI) CMOS technology, in which both the piezoresistive microcantilever array and its on-chip signal processing circuit were fabricated on the device layer of an SOI wafer. This monolithic integration process takes advantage of a silicon-based microcantilever with a high gauge factor and SOI CMOS with low parasitic, latch-up, and leakage currents compared with bulk silicon CMOS circuits. The excellent performance of the developed integrated microcantilevers was verified by detecting human IgG, abrin, and SEB, and the results prove that the integrated microcantilevers offer great potential for use in label-free, real-time and high-sensitivity detection.

## Results and discussion

### Design of the monolithically integrated microcantilever

**Fig. 1:**
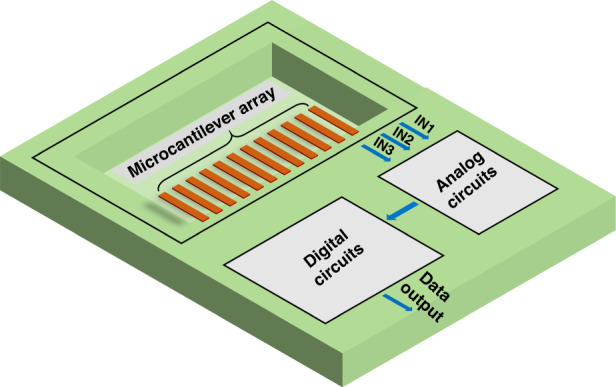
The overall architecture of the monolithically integrated microcantilever. The monolithically integrated microcantilever consists of a microcantilever array, analog circuits and digital circuits

Figure 1 shows the overall architecture of the proposed integrated microcantilever sensors, which are composed of twelve piezoresistive microcantilevers and a signal processing circuit. Four microcantilevers with embedded piezoresistors form a sensor in a Wheatstone bridge configuration; therefore, three integrated piezoresistive microcantilever sensors were designed on a chip. The signal processing circuits execute amplification and analog-to-digital conversion of output signals from the three channels and then transfer the signals to serial peripheral interface circuits for digital signal output.

### Design of the piezoresistive microcantilever sensor

**Fig. 2:**
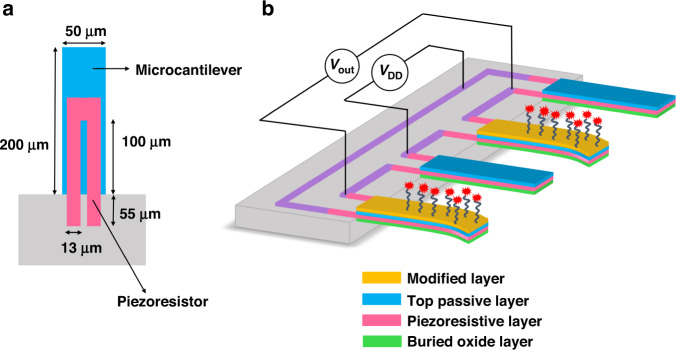
Schematic diagram of the microcantilever. **a** Schematic diagram of the U-shape piezoresistor. **b** Schematic diagram of the piezoresistive microcantilever sensor

Three sets of Wheatstone bridges were implemented by twelve piezoresistive microcantilevers with identical dimensions on the integrated microcantilever chip. Based on the optimized results of our group^19,29^, the piezoresistive microcantilever was rectangular with dimensions of 200 μm long, 50 μm wide, and 1-μm-thick. According to finite element analysis, the maximum stress is concentrated at the root of the microcantilever; therefore, the piezoresistor was arranged in a U-shape with one leg dimension of 100 μm × 13 μm and embedded at the fixed end of the microcantilever to ensure a high sensitivity. In addition, the U-shape piezoresistors are longitudinally oriented with respect to the microcantilever axis, as shown in Fig. 2a. To decrease the thermal drift and increase the sensitivity of the piezoresistive microcantilever, the resistance of the piezoresistor was optimized to be 8 kΩ, which was formed by boron ion implantation with a doping dose of 3 × 10^14^ cm^−2^.

Four piezoresistive microcantilevers were configured as a DC-bias Wheatstone bridge (actually a quasihalf bridge) to measure weak changes in resistance, two of which serve as reference microcantilevers, and the remaining two gold-covered microcantilevers are measurement microcantilevers, as shown in Fig. 2b. The differential design can reduce the effects of mechanical vibration and variation in the ambient temperature or humidity on the sensor, as well as compensate for resistance (but not sensitivity) changes with temperature^30,31^. For future applications, a specific compensation circuit can be applied for temperature-based sensitivity compensation. The reference microcantilevers consist of a 400 nm buried oxide layer, a 340 nm single-crystalline silicon piezoresistive layer, and a 300 nm top SiO_2_ passive layer, whereas the measurement microcantilevers have an extra modified layer of 10/50 nm Ti/Au for immobilizing probe molecules. Once the binding process between the probes and the target molecules occurs, surface stress is generated on the measurement microcantilevers. The surface stress induces measurement microcantilever bending, thus causing a resistance change in the embedded piezoresistors. The detected binding process is converted into a differential voltage signal by the Wheatstone bridge, which can be described as:

1 $$V_{\rm{out}} = \frac{1}{2}V_{\rm{DD}}\frac{{\Delta R}}{R}$$

where *V*_DD_ is the supply voltage, *R* is the initial resistance of the piezoresistor, and Δ*R* is the resistance change caused by the binding process.

### Design of the signal processing circuit

**Fig. 3:**
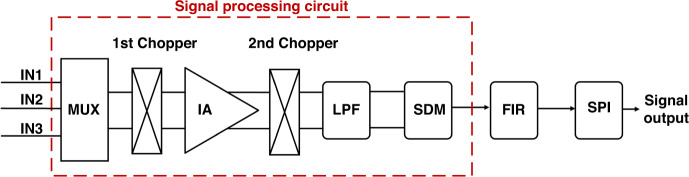
Schematic diagram of the signal processing circuit. The signal processing circuit is composed of the MUX, IA, LPF, SDM, FIR and SPI

As shown in Fig. 3, the on-chip signal processing circuit consists of a time-division multiplexer (MUX), a chopper instrument amplifier (IA), a low-pass filter (LPF), a sigma-delta modulator (SDM), a finite impulse response filter (FIR), and a serial peripheral interface (SPI). The differential signals from three-channel microcantilevers are first sent to the time-division MUX composed of complementary CMOS switches, which is controlled by a clock to select a signal from the different sensors and transmit it to the IA.

To detect the weak output signal of the microcantilever sensor with high SNR, chopper technology was introduced in the IA for low-noise signal amplification. A fully differential two-stage op-amps topology was adopted to amplify the selected signals from the MUX. The first stage includes two preamplifiers and corresponding feedback resistors to achieve high input impedance, and the second stage is composed of a differential amplifier and its feedback resistors to provide wide output swing. Meanwhile, the gain-adjustable structure was designed to ensure that the signal from the microcantilever is amplified to the full swing of the SDM to enhance the overall signal path SNR. The simulated results show that a maximum gain of 136 and an equivalent input noise of only 7.85 nV/√Hz at 10 Hz bandwidth can be obtained. According to our group’s previous report^19^, the intrinsic noise of the piezoresistive microcantilever was 51.7 nV/√Hz at a 10 Hz bandwidth and 3 V biased voltage, which was higher than that of the designed IA, demonstrating the IA to be a low-noise system.

To realize a signal readout lower than 1 μV for the microcantilever, a 24-bit signal conversion accuracy is required for the sigma-delta analog-to-digital converter (Σ-Δ ADC). Therefore, a 2nd-order single-loop switched-capacitor modulator with a cascade-of-integrators feedback architecture was employed due to its insensitivity to parameter variations compared with a continuous-time modulator. The FIR was used to convert the pulse width modulation signal from the SDM into a 24-bit digital signal and filter out high-frequency quantization noise completely. Therefore, a 61-order architecture was adopted with a cutoff frequency at 2 Hz. The simulated results show that the SNR of the SDM is greater than 100 dB, and the effective number of bits (ENOB) is more than 16.

### Layout design of the monolithically integrated chip

The monolithically integrated microcantilever chip is divided into three modules in the layout: a microcantilever sensor module, an analog circuit module, and a digital circuit module. To ensure that the sensor signal was exported with high SNR, the following key design points were adopted in the layout design of the integrated chip: (a) All the piezoresistors, PMOS, and NMOS devices of the signal processing circuit were designed on the device layer of an SOI wafer with silicon oxide as insulation; (b) the first metal layer in SOI CMOS was adopted as electrical connections between the piezoresistors in the Wheatstone bridge, and the second and third metal layers of SOI CMOS were used as the interconnection lines between the Wheatstone bridges and the MUX, with all the interconnection lines were arranged symmetrically to minimize the initial offset of the Wheatstone bridge; (c) to avoid mutual interference between the sensor, analog, and digital signals, the power supply and ground of the three modules were separated from each other, and power rails were also designed separately for the three modules, with decoupling capacitors between the power and ground placed in the empty area to reduce the impact of voltage level variation; (d) to protect the CMOS circuit from electrostatic breakdown, the grounded-gate NMOS and gate-drain PMOS were used as the electrostatic protection devices around the pads; (e) to ensure the subsequent MEMS processes and biomolecule detections in PBS, the circuits and the sensors were separated by a certain distance, and all the pads were placed at the side of the circuits. Finally, the size of the finished integrated microcantilever chip is 4.26 mm × 3.86 mm, which includes a reaction well beneath the microcantilever array for enabling biomolecular detection.

**Fig. 4:**
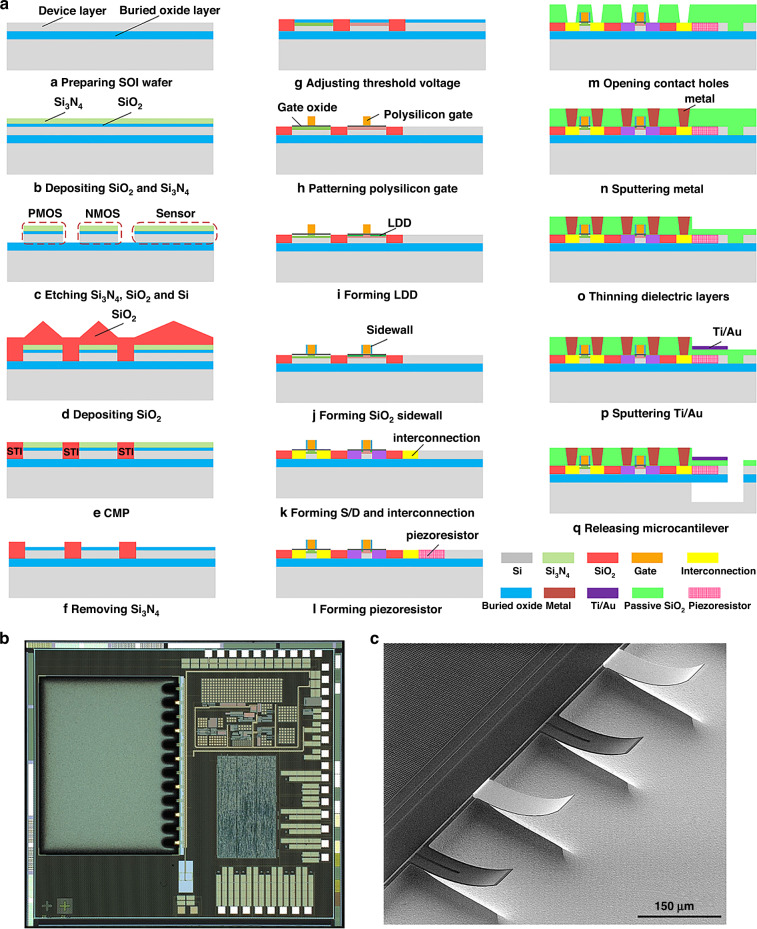
Fabrication of the integrated microcantilever. **a** The developed monolithic integration process. **b** Micrograph of the fabricated integrated microcantilever. **c** SEM photograph of the microcantilever array

Since the buried oxide layer of the SOI wafer can isolate the microcantilever from the substrate, reduce the leakage current, and obtain lower noise^32^, it was adopted to fabricate the integrated microcantilevers. A 0.15 μm standard PD-SOI CMOS process with one-layer polysilicon and four-layer metals was utilized to fabricate the integrated microcantilevers. Figure 4a shows a schematic diagram of the basic fabrication steps in one-layer polysilicon and one-layer metal. The micrograph of the fabricated monolithically integrated microcantilever is presented in Fig. 4b. The scanning electronic microscopy (SEM) image in Fig. 4c shows that the released microcantilever array is slightly bent, which is caused by the residual stress in the multilayer stacked structure. However, the impact on microcantilever performance can be compensated by the same design of four microcantilevers and the Wheatstone bridge configuration.

### Performance of the monolithically integrated microcantilever

During detection, the digital signals from the integrated microcantilever sensor were collected by a commercial FPGA via the SPI. To monitor the output voltage of the integrated microcantilever from the FPGA, software was developed to process digital signals and display real-time voltage on a laptop. The relationship between the digital signal and the actual output voltage of the microcantilever was calibrated with a digital output resolution of 2.9 nV/LSB.

**Fig. 5:**
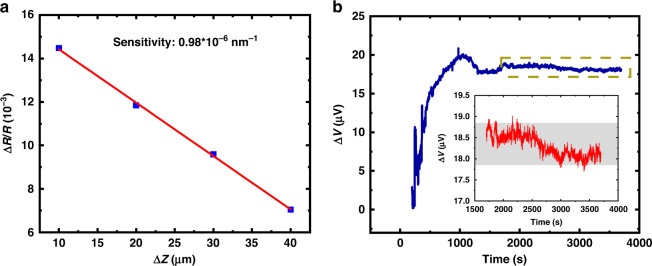
Performance of the integrated microcantilever. **a** The test curve of deflection sensitivity. **b** The test result of output stability

The deflection sensitivity of the microcantilever is defined as ∆*R*/*R*·∆*z*^−1^, where ∆R/R is the relative change in resistance of a single microcantilever and Δ*z* is the vertical displacement change at the free end of the single microcantilever after applying a force. During the test, the integrated microcantilever was fixed on a probe stage, and the probe applied a force to the free end of the microcantilever with 10 µm steps, thereby changing the resistance of the piezoresistor. The resistance was measured by a 6½ digital multimeter (Agilent, 34401A). The relative change in resistance as a function of the vertical displacement of the microcantilever is shown in Fig. 5a, in which the deflection sensitivity was calculated as 0.98 × 10^−6^ nm^−1^ by fitting the slope of the curve. The deflection resolution, defined as the minimum detectable deflection, was calculated to be 0.62 nm at a bandwidth of 1000 Hz and a biased voltage of 3 V^19,33^.

Since the biosensing experiments are performed in PBS buffer, the stability in PBS (pH = 7.2~7.4, 0.01 M) was tested at room temperature. During the experiment, the integrated microcantilevers were immersed into an external detection well with a volume of 1000 μL to ensure that the integrated microcantilevers were completely immersed. However, to prevent short circuits among the bonding wires, the volume of PBS solution injected into the detection well is usually slightly less than 1000 μL. Figure 5b depicts the stability test curve. It is worth noting that the output voltage increased rapidly at the beginning, which can be attributed to the self-heating effect of the piezoresistor embedded in the microcantilever, as well as the difference in thermal deformation between the measurement microcantilevers and the reference microcantilevers due to the different thermal expansion coefficients of Au and SiO_2_. After the initially rapid increase, the output voltage reached a dynamic equilibrium. At this point, the temperature cross sensitivity played a minor role, while the intrinsic noise of the piezoresistors played a dominant role in determining SNR. It can be seen from the inset that an output voltage fluctuation of 1 μV at a 3 V bias voltage was attained after approximately 1700 s, demonstrating that the proposed integrated microcantilevers are suitable for high-sensitivity detection of trace biological substances.

### Performance of the signal processing circuit

The performance of the signal processing circuit was measured by a chip-testing system (ADVANTEST V93000), and the measured results are summarized in Table 1. Table 1 shows that the maximum gain of the IA is 134.97, which is close to the theoretical value of 136. The measured input offset current is only 0.623 nA, verifying that the leakage current can be well suppressed using PD-SOI CMOS technology. An input offset voltage of 3.72 μV is acquired, proving that the designed amplifier is suitable for high-precision applications, as expected.

**Table 1:** The measured results

CMOS circuits	Maximum gain	134.97
	Chopper frequency	30.24 kHz
	OSC frequency	962.1 kHz
	PSRR	−105.65 dB/−121.58 dB
	CMRR	−109.41 dB/−102.46 dB
	Input offset voltage	3.72 μV
	Input offset current	0.623 nA
	SNR	70 dB
	ENOB	11 bits
	Bandwidth	6 Hz

With the input of the SDM maintained, the performance of the SDM cannot be characterized accurately. The measured ENOB of the SDM is only 11 bits, despite that the actual resolution on the voltage signal demonstrates that the simulated result of 16 bits is achievable. The SNR is measured to be 70 dB because the supply voltage fluctuation and parasitic resistance of the test board degrade the SNR of the SDM.

The above measurement results demonstrate that the integrated microcantilevers have high sensitivity by utilizing single-crystalline silicon as the piezoresistors, good signal stability achieved by the silicon island isolation structure, and high PSRR, CMRR, and low input offset voltage for the on-chip signal processing circuit, making the system applicable for biomolecule detection. The PD-SOI CMOS-based monolithically integrated technology takes advantage of low parasitic capacitance, leakage current, latch-up effects, etc., compared with bulk silicon CMOS. In addition to fabricating the integrated microcantilever sensor, this technique can also be applied to process other piezoresistive integrated MEMS devices.

To demonstrate the detection capability of the integrated microcantilever sensors with high SNR, detection of human IgG, abrin and SEB was performed.

### Detection of human IgG

Before detection experiments, the integrated microcantilever needs to be functionalized to immobilize the probes on the Au surface of the measurement microcantilevers. A biotin-avidin system (BAS) method was utilized to achieve surface functionalization of the integrated microcantilevers because of its high affinity and multistage amplification effect. After surface functionalization, a sample solution of 200 μg/mL biotinylated goat anti-human IgG was injected into the detection well and incubated for 1 h to ensure that the Au surface of the measurement microcantilevers could adequately immobilize antibodies with a low detection limit. After multiple cleanings, the functionalized microcantilever sensors were immersed into the detection well of 870 μL PBS until a stable output voltage was achieved. Then, human IgG at final concentrations of 1.0 ng/mL, 2.0 ng/mL, and 5.7 ng/mL was applied to the detection well.

**Fig. 6:**
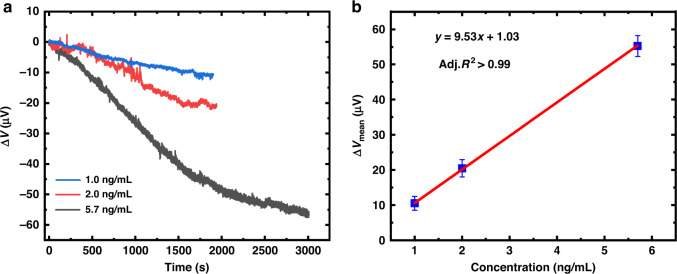
The real-time response of the integrated microcantilever sensors. **a** The measured results of human IgG at different concentrations. **b** The saturated voltage change versus IgG concentrations

The real-time response curves of the integrated microcantilever sensors exposed to human IgG are illustrated in Fig. 6a. The output voltage of the integrated microcantilever sensors changed accordingly when human IgG was injected into the detection well at different concentrations. The response magnitudes increased with increasing human IgG concentrations, and all the responses reached relatively saturated states after a certain time. This effect is attributed to the fact that more human IgG molecules bind to the immobilized antibodies on the measurement microcantilevers at high concentrations, resulting in greater surface stress. The saturated voltage change as a function of human IgG concentration is shown in Fig. 6b, indicating a good linear relationship. In addition, the same concentration measurements were performed 3–5 times by different microcantilever biosensors since the bound human IgG was difficult to remove from the surface of the microcantilever biosensor. The results suggest that the curves present similar response trends at the same concentration, verifying that the integrated microcantilever sensors have good repeatability. The voltage changes caused by human IgG binding with antibodies were 10.4 ± 2.0, 20.5 ± 2.5 and 55.2 ±3 .0 μV, corresponding to concentrations of 1.0 ng/mL, 2.0 ng/mL, and 5.7 ng/mL, respectively, and the standard deviations are indicated in Fig. 6b in the form of error bars.

### Detection of abrin

**Fig. 7:**
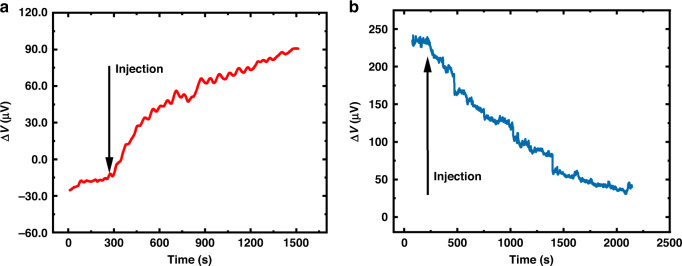
The real-time response of the integrated microcantilever sensors. **a** The measured results of abrin. **b** The measured results of SEB

Abrin is a highly toxic protein with a relative molecular mass of 65 kDa^34^ that is composed of an A-chain and a B-chain. The estimated lethal oral dose of abrin in humans is 0.1–1 μg/kg^35,36^. Unintentional abrin ingestion can quickly cause irreparable organ failure and even death, so rapid, label-free, and sensitive detection of abrin is desired. After the aforementioned functionalization steps, a 10 μM aptamer solution of abrin was injected into the detection well and incubated for 1 hour to ensure that enough aptamers were immobilized on the Au surface of the measurement microcantilevers. The functionalized microcantilever aptasensor was immersed in 810 μL PBS solution until the output signal reached a steady state. Then, a 40 μL abrin solution with a concentration of 200 ng/mL was trickled into the detection well. The real-time detection result of abrin with a final concentration of 9.4 ng/mL is shown in Fig. 7a. The output voltage changed immediately once the aptamers bound with abrin molecules. This can be explained by the fact that the binding process leads to a change in the conformation of the aptamers, generating surface stress on the measurement microcantilevers^37^. After 1200 s, most of the abrin molecules have bound to the probes immobilized on the surface of measurement microcantilevers, thus slowing down the binding speed and showing a tendency of saturation. At this condition, the output voltage has changed by 108.9 μV. According to the output stability measurement result in Fig. 5b of 1 μV voltage fluctuation, the LOD of abrin was calculated as approximately 86 pg/mL.

### Detection of SEB

SEB is a highly heat-stable pathogen with a relative molecular mass of 28.336 kDa, which is derived from gram-positive *Staphylococcus aureus* bacterium^38,39^. While death rarely occurs after SEB poisoning, severe nausea, vomiting, and gastrointestinal cramps typically occur after ingestion^14^. Hence, there is an urgent demand for a simple, rapid, and sensitive method to detect SEB. Using a similar detection method with abrin, a 10 μM aptamer solution of SEB was added into the detection well to complete the surface functionalization of the measurement microcantilevers. The functionalized microcantilever aptasensor was immersed into the detection well of 810 μL PBS to obtain a stable output baseline. After stabilization, a 40 μL SEB solution with a final concentration of 9.4 ng/mL was trickled into the detection well. The detection result is shown in Fig. 7b, proving that the prepared integrated microcantilever aptasensor has a significant response to SEB. This can be attributed to the formation of aptamer–SEB conjugates, resulting in the measurement microcantilevers bending. The trends of the voltage change are opposite when detecting SEB and abrin. This occurs because the initial bending direction of the microcantilevers changed from upward to downward in detecting SEB after the microcantilever sensor was completely immersed into the PBS solution, which is caused by the surface tension of the liquid. However, when the target molecules bind with the aptamers immobilized on the measurement microcantilever, surface stress is generated that causes the microcantilever to bend upward^40,41^. Thus, the stress increased in detecting abrin, while the stress decreased in detecting SEB, leading to opposite trends in the detection curves for SEB and abrin. We can observe that the output voltage reaches saturation after approximately 1900 s, and the changed voltage was 196.2 μV. Therefore, the LOD of SEB was estimated to be 48 pg/mL based on the output stability measurement result in Fig. 5b of 1 μV voltage fluctuation. Compared with an integrated microcantilever working in dynamic mode^27^, the LOD of 48 pg/mL in our work is at the same level for detecting HBV.

### Three-channel verification

**Fig. 8:**
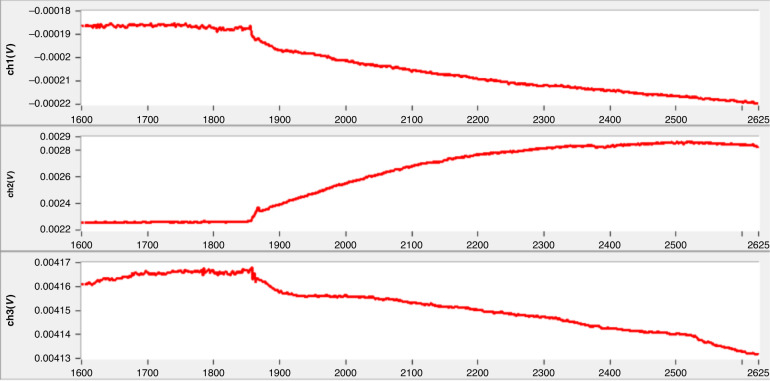
The three-channel test results. Real-time response of the first, the second and the third channels to SEB with a concentration of 200 ng/mL from top to bottom

Because all the integrated microcantilevers were designed in the same reaction well, the three-channel function can only be verified with a single biomolecule detection. In this test, SEB was used for three-channel verification, and the functionalization steps for the three-channel detection of SEB were the same as those for single-channel detection. The functionalized microcantilever aptasensors were placed into PBS solution until the output signals from the three channels reached dynamic equilibrium. Then, a 40 μL SEB solution with a final concentration of 200 ng/mL was applied into the detection well. The dynamic response curve for the three-channel detection of SEB is shown in Fig. 8.

The three channels of microcantilever aptasensors responded simultaneously, and the output signals of all channels showed a significant change induced by molecular adsorption. The output voltage in different channels changed in the opposite direction. This is possibly attributed to the initial bending direction of the microcantilevers in different channels changing from upward to downward due to the surface tension of the liquid after the microcantilever sensors were immersed in the PBS solution. In addition, if the initial bending was severe, the microcantilevers likely stuck to the triangular bumper structure (a residual from the cantilever release etching process) when immersed in the PBS solution. The three-channel detection of SEB was completed within approximately 800 s, which is much faster than the single-channel detection of SEB with a concentration of 9.4 ng/mL and is attributed to collision theory^42^. When the concentration is higher, the molecular collision between aptamers and target molecules is more intense, so the saturation time is shorter.

## Conclusion

In this paper, we propose a monolithically integrated microcantilever consisting of a piezoresistive microcantilever array and an on-chip signal processing circuit to present a monolithic integration process using PD-SOI CMOS technology. All performance test results demonstrate that the design and fabrication process of the integrated microcantilever are successful and can satisfy the requirements of high-sensitivity detection of biomolecules. Detection of human IgG, abrin, and SEB at different concentrations was realized in PBS solution using the integrated microcantilever aptasensors as functionalized by the BAS method. A favorable linear response and multichannel detection of the integrated microcantilever aptasensors were verified, and an LOD of 48 pg/mL was achieved when detecting SEB. The developed monolithically integrated microcantilever aptasensors are capable of biomolecule detection with the advantages of high SNR, portability, and label-free detection. Furthermore, the SOI CMOS-based integration process has wide applicability and can be applied to fabricate other piezoresistive integrated MEMS sensors.

## Materials and methods

### Fabrication of the integrated microcantilever

To increase the sensitivity of the microcantilever, SOI wafers with a 340-nm-thick p-type single-crystalline silicon in the [110] orientation and a 400-nm-thick buried oxide layer beneath were adopted to fabricate the integrated microcantilevers. The single-crystalline silicon device layer serves both as the piezoresistive layer of the sensors and the active region of the SOI CMOS circuits. The buried oxide layer is used as both the bottom insulation layer and the etch self-stop layer of the microcantilever, which can greatly simplify the release process of the integrated microcantilever.

According to the thickness of the device layer and its depletion, the SOI CMOS process is divided into fully depleted (FD)-SOI CMOS technology and partially depleted (PD)-SOI CMOS technology with 100 nm as a cutoff thickness. Therefore, PD-SOI CMOS technology was adopted to implement the monolithically integrated microcantilever. To address the kink effect in PD-SOI CMOS, BTS-shape and H-shape body contacts were designed, which allow the hole charges in the body region to flow into the source end.

A 0.15 μm standard PD-SOI CMOS process with one-layer polysilicon and four-layer metals was expanded to fabricate the integrated microcantilevers, and the expanded processes include the formation of the piezoresistor, thinning of the top dielectric layer on the microcantilevers, deposition and patterning of the Ti/Au film on the measurement microcantilevers, and release of the microcantilevers. The other plane processes of the microcantilevers were completed synchronously with the SOI CMOS process. Figure 4a shows a schematic diagram of the basic fabrication steps in one-layer polysilicon and one-layer metal, which can be described as follows:

(a)~(e): A thin layer of silicon dioxide (SiO_2_) was generated by thermal oxidation, and a layer of silicon nitride (Si_3_N_4_) was deposited using low-pressure chemical vapor deposition (LPCVD) for the etching mask. Then, Si_3_N_4_, SiO_2_, and a single-crystalline silicon device layer were etched away to form silicon island isolation structures for circuits and sensors. Next, the SiO_2_ insulating layer was deposited to achieve electrical isolation between the island isolation structures, followed by the chemical mechanical polishing (CMP) process to form shallow trench isolation (STI).

(f)~(g): The N well was formed for PMOS devices, and an N+ ring was also generated around the piezoresistor to simultaneously reduce the leakage current. The threshold voltage of the NMOS and PMOS devices was adjusted by low-energy and low-dose ion implantation.

(h): After the gate oxide was formed by thermal oxidation with its thickness adjusted according to the designed threshold voltage, a layer of polysilicon was deposited by LPCVD, followed by ion implantation to adjust the work function of the polysilicon with a doping dose of 8 × 10^15^ cm^−2^ and an energy of 70 keV. After that, the polysilicon gate was patterned.

(i)~(k): Lightly doped drain-source (LDD) structures were implemented with a doping dose of 2.5 × 10^13^ cm^−2^ at 30 keV to suppress the short channel effect, in which a SiO_2_ sidewall was formed to block ion lateral scattering during source/drain doping. The source and drain regions were formed by boron and phosphor ion implantation and defining the heavily doped interconnection lines simultaneously. The body contact region was implanted during this process.

(l): The resistance of the piezoresistors was adjusted by boron ion implantation. Due to the lack of a suitable doping dose in the PD-SOI CMOS process, an additional lithography step was added, followed by boron ion implantation with a doping dose of 3 × 10^14^ cm^−2^ and an energy of 50 keV to implement the approximately 8 kΩ piezoresistor resistance.

(m)~(n): After depositing the dielectric layer, the contact holes were opened. Then, four-layer metals were sputtered and patterned to form interconnection lines and pads.

(o): Since the thickness of the dielectric layers, which include dielectric layers between metals and the passivation layer above the circuits, is as high as 3.3 μm, leading to a sharp drop in sensitivity, the SiO_2_ dielectric layer above the sensor was thinned to approximately 300 nm by dry etching, ensuring that the overall thickness of the microcantilever is approximately 1 μm.

(p): In the penultimate step, a 10/50 nm Ti/Au film was sputtered by physical vapor deposition and patterned on the surface of measurement microcantilevers by a lift-off method to form a modified layer, which facilitates the binding process between the probes and the target molecules.

(q): Finally, a hybrid process of anisotropic and isotropic dry etching instead of KOH wet etching was adopted to release the microcantilevers, which avoids potassium contamination that may have an influence on the output signal stability of the integrated microcantilevers. After the last lithography with a 10-μm-thick photoresist as a mask, the top passivation layer and buried oxide layer above the reaction well were completely etched using reactive ion etching until the Si substrate was exposed. Then, the Si substrate was etched 10 μm using anisotropic deep reactive ion etching (DRIE), followed by isotropic DRIE for approximately 25 μm, until the microcantilever arrays were fully released, and the buried oxide layer served as the bottom etch self-stop layer of the microcantilever. To reduce microcantilever fracture caused by stress introduced by excessive energy, the ion etching power was reduced to 1200 W during the etching process.

### Surface functionalization of integrated microcantilevers

Prior to surface functionalization, the integrated microcantilevers were preconditioned using oxygen plasma for 5 mins to remove the residual photoresist. Next, the integrated microcantilevers were put into piranha solution (75%H_2_SO_4_, 25%H_2_O_2_) for 15 mins to remove organic contamination.

After the pretreatment processes, the cleaned microcantilevers were incubated in 5 mg/mL DDPA (3,3′-dithiopropionic acid) for one hour to provide carboxyl groups, while Au–S covalent bonds were formed on the Au surface of the measurement microcantilevers. Subsequently, the microcantilevers were treated in a 5 mg/mL EDC (1-ethyl-3-3-dimethylaminopropyl-carbodiimide hydrochloride)/NHS (N-hydroxy succinimide) mixture (*v*/*v* = 2:3) for 30 min to transform the carboxyl groups into active esters. Then, 300 μg/mL diluted streptavidin was added to the detection well for an hour incubation.
